# Generation of Regulatory T Cells From Human Memory CD4^+^T Cells by Upregulation of Naked Cuticle Homolog 2

**DOI:** 10.1002/eji.70018

**Published:** 2025-08-05

**Authors:** Jiajun He, Kristy Ou, Michael Schmueck‐Henneresse, Edgar Specker, Jérôme Paul, Marc Nazare, Jens Peter von Kries, Julia K. Polansky, Alf Hamann, Stefan Frischbutter

**Affiliations:** ^1^ Institute of Allergology Charité – Universitätsmedizin Berlin, Corporate Member of Freie Universität Berlin and Humboldt‐Universität zu Berlin Berlin Germany; ^2^ Fraunhofer Institute for Translational Medicine and Pharmacology ITMP, Allergology and Immunology Berlin Germany; ^3^ Department of Dermatology The Affiliated Hospital of Southwest Medical University Luzhou China; ^4^ BIH Center for Regenerative Therapies (BCRT) T Cell Epigenetics Berlin Institute of Health at Charité ‐ Universitätsmedizin Berlin Berlin Germany; ^5^ BIH Center for Regenerative Therapies (BCRT) Berlin Institute of Health (BIH) At Charité ‐ Universitätsmedizin Berlin Berlin Germany; ^6^ Berlin Center for Advanced Therapies Charité ‐ Universitätsmedizin Berlin, Corporate Member of Freie Universität Berlin, Humboldt‐Universität zu Berlin, and BIH Berlin Germany; ^7^ Chemical Biology Platform Leibniz‐Forschungsinstitut Für Molekulare Pharmakologie (FMP) Berlin‐Buch Germany; ^8^ Immuno‐Epigenetics German Rheumatism Research Centre (DRFZ) Berlin Germany; ^9^ Department of Rheumatology and Clinical Immunology Charité ‐ Universitätsmedizin Berlin Berlin Germany

## Abstract

Regulatory T cells are indispensable for immune homeostasis and tolerance to self‐antigens and allergens. The imbalance between immune responses and tolerance causes allergic and autoimmune diseases. A promising therapeutic strategy is to support immune tolerance by converting conventional T cells into suppressive regulatory T cells with small molecular weight compounds, an area that is underexplored. Here, we report the identification, characterization, and validation of a novel quinoxaline derivative (IFA005) that converts human memory CD4^+^T cells into suppressive Foxp3‐expressing Tregs in vitro. Mechanistically, IFA005 regulated the expression of naked cuticle homolog 2 and impaired the phosphorylation of glycogen synthase kinase‐3β, which led to the degradation of β‐catenin and thus blocked the Wnt‐β‐catenin pathway. Our findings indicate that IFA005 could be a promising candidate for inducing immune tolerance by converting effector T cells into suppressive Treg cells through the inhibition of the Wnt‐β‐catenin pathway.

## Graphical abstract

This study discovered the small molecule 9‐methoxy‐6‐methylindolo[3,2‐b] quinoxaline (IFA005) as a potent immunomodulator capable of converting human memory CD4⁺ T cells into functional Foxp3⁺ regulatory T cells. IFA005 suppresses Th2 cytokines (IL‐4, IL‐5, and IL‐13) and promotes the iTreg phenotype by inhibiting Wnt/β‐catenin signaling through NKD2 upregulation. These findings support small‐molecule‐mediated Wnt/β‐catenin blockade as a promising strategy to induce immune tolerance in allergic and chronic inflammatory diseases.

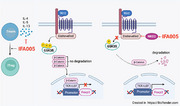

## 1 Introduction

Autoimmunity, whether organ‐specific or systemic, results from a loss of immunologic self‐tolerance. The worldwide prevalence of autoimmune diseases (including allergies) is rapidly increasing, mainly due to environmental and socioeconomic influences, requiring novel therapeutic options [1]. Regulatory T cells (Tregs), a specialized subset of T lymphocytes, possess immunosuppressive activities, indispensable for establishing immune tolerance against harmless environmental antigens or self‐antigens [2, 3, 4, 5]. The transcription factor forkhead box P3 (Foxp3) controls the development and suppressive activity of Treg cells [6, 7]. Three types of Treg cells can be distinguished: thymus‐derived Tregs (tTregs), which are originally generated in the thymus, peripherally derived Treg cells (pTregs), and in vitro induced Treg cells (iTregs), which are induced from naïve T cells [8]. Treg cells have valuable therapeutic potential in chronic autoimmune conditions [9], and the first promising therapeutic effects were recently demonstrated in human autoimmune diseases [10, 11] and preclinical models of allergies [12, 13, 14].

Currently, three main strategies based on Tregs are explored in the development of treatments for autoimmune and allergic diseases [5]. The first is the ex vivo expansion of tTregs and subsequent adoptive cell therapy (ACT) or the generation and transfer of chimeric‐antigen‐receptor Tregs (CAR‐Tregs). ACT is used mostly in clinical trials on solid organ transplant, graft‐versus‐host disease, and selected autoimmune diseases such as type 1 diabetes and systemic lupus erythematosus [15]. The limitations of this approach include the need to generate functional cells in sufficient numbers and the risk of infections and cancer owing to the potent suppressive activity of tTregs. CAR‐Treg can be generated in larger quantities, but high costs and safety concerns must be considered. The second Treg–based therapeutic approach is the in vivo enhancement of tTregs via biologics or synthetic molecules. For example, low‐dose IL‐2 is an emerging treatment strategy for autoimmune diseases such as rheumatoid arthritis and type 1 diabetes [16]. Its downsides include the short duration of effects, injection site reactions [17], and the risk of causing immunosuppression and cancers [18]. While the former two approaches are based on targeting tTregs, the third strategy is to convert memory T cells into pTregs or iTregs, which has been attempted with retinoic acid [19], TGF‐β, and short‐chain fatty acids [20]. However, these approaches failed to transform pathogenic memory T cells into Tregs.

Alternatively, small molecules that convert conventional T cells, especially memory T cells, into Tregs in vivo might be another promising strategy, since memory T cells promote inflammation and autoimmunity. The feasibility and proof of concept of this approach in inflammatory and allergic conditions have recently been reported [21, 22]. However, as of now, there is a lack of small molecules that directly promote the conversion of memory T cells into iTreg cells.

To address this unmet need, we conducted a high‐throughput phenotypic screen of a small molecule library, employing a Foxp3‐eGFP reporter system, to identify compounds that induce Foxp3 upregulation in CD4^+^T cells. Here, we report the identification, characterization, and validation of a novel quinoxaline derivative that converts human primary memory CD4^+^T cells into suppressive Foxp3‐expressing Tregs in vitro.

## 2 Results

### 2.1 IFA005 Converts Human Conventional CD4^+^T Cells into Foxp3‐expressing Tregs

**FIGURE 1:**
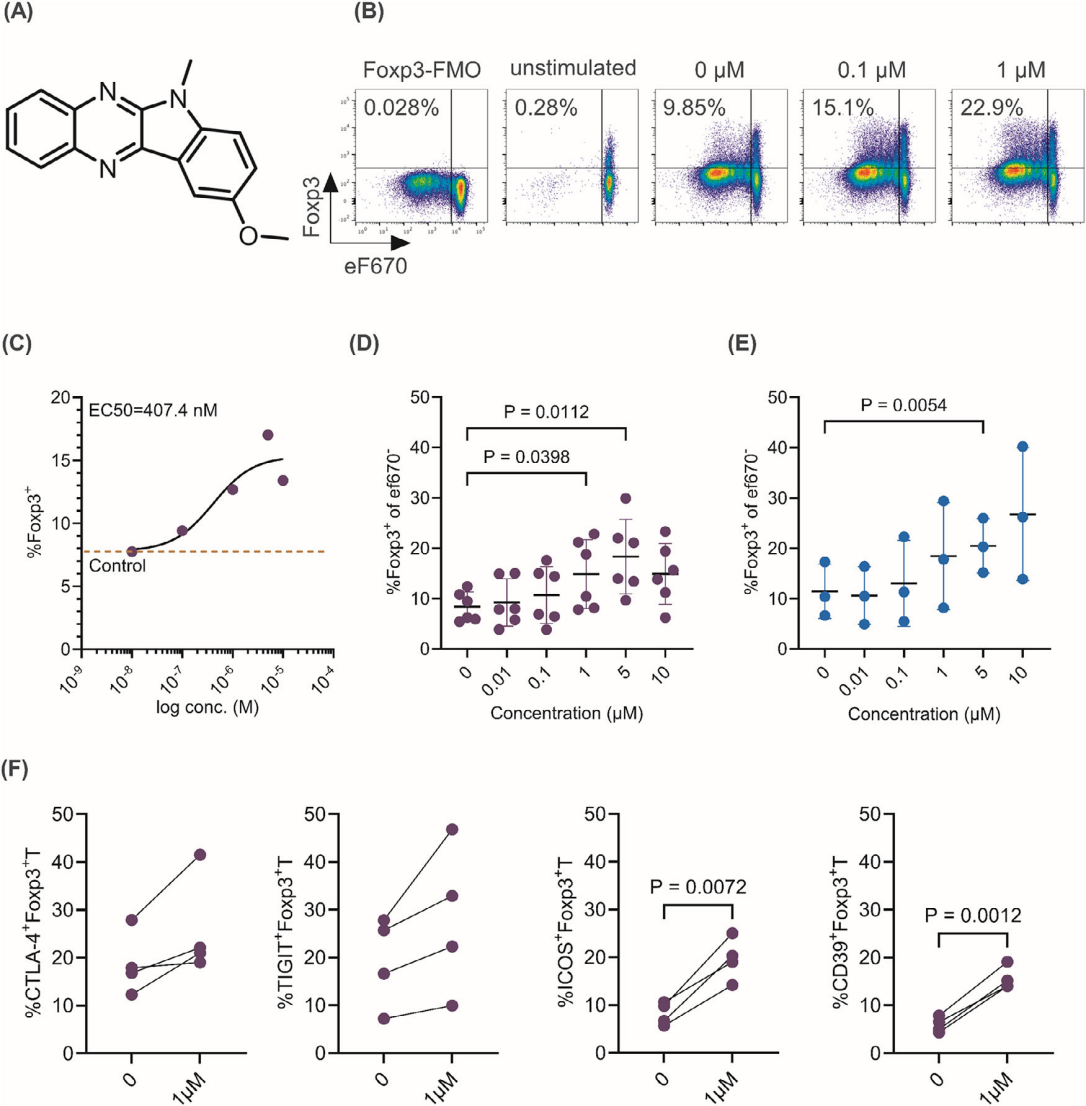
IFA005 induces Foxp3 expression in human primary CD4^+^T cells. (A) The chemical structure of 9‐methoxy‐6‐methylindolo[3,2‐b] quinoxaline (IFA005). (B‐E) Human primary memory CD4^+^CD45RO^+^T cells (Tmem) or naive CD4^+^CD45RA^+^T cells (T_N_) were stimulated with plate‐bound anti‐CD3/CD28 mAb in the presence of indicated concentrations of IFA005 for 5 days, and cell proliferation was monitored with cell proliferation dye eFluor 670 (eF670). (B) Gating of Foxp3^+^ cells among memory CD4^+^T cells, according to Foxp3‐FMO. (C) Dose response curve of Foxp3 induction, using four‐parameter logistic analysis to calculate EC_50._ Each dot represents the mean of 6 individual experiments. The dashed line is a reference line of the Foxp3 induction showing Foxp3 expression without IFA005 (*n* = 6). (D) The percentage of Foxp3 expression in proliferated Tmem (*n* = 6). (E) The frequency of Foxp3 in proliferated T_N_ (n = 3). (F) Tmem were stimulated as mentioned above in the presence or absence of 1 µM of IFA005 for 5 days. The frequency of CTLA4^+^Foxp3^+^, TIGIT^+^Foxp3^+^, CD39^+^Foxp3^+^ and ICOS^+^Foxp3^+^ cells among CD4^+^T cells is shown (*n* = 4). All data are presented as mean ± SD. One‐way ANOVA was applied for statistics in (D, E), paired *t*‐test was applied for statistics in (F). Data points in graphs (D–F) represent individual experiments.

We developed a cellular assay system based on primary murine Foxp3‐eGFP‐expressing T cells and used high‐throughput flow cytometry to screen a library of 41,184 compounds to identify small molecular compounds that induce Foxp3 expression in conventional CD4^+^T cells (Tconv), as previously described in chapter VIII.12 [23]. After two rounds of hit validation in the murine Foxp3‐eGFP reporter cells, we selected one compound, 9‐methoxy‐6‐methylindolo[3,2‐b] quinoxaline (IFA005, Figure 1A), for further validation in primary human CD4^+^ naïve (CD45RA^+^) and memory (CD45 RO^+^) Tconv. IFA005 significantly and dose‐dependently (EC_50_ = 407.4 nM, Figure 1C) upregulated Foxp3 expression in memory Tconv cells at concentrations of 1 µM (*p* = 0.04) and 5 µM (*p* = 0.01) (Figure 1D); the gating strategy is shown in Figure 1B. In naïve Tconv, Foxp3 expression was significantly upregulated in the presence of IFA005 at 5 µM (*p* = 0.005) (Figure 1E). Of note, Foxp3 upregulation occurred in the absence of antigen‐presenting cells (APC) or transforming growth factor β (TGF‐β), which have been described to be involved in the induction of Foxp3 from Tconv in the thymus or in the periphery [24]. Furthermore, we assessed the toxicity of IFA005 by measuring cell viability and proliferation. IFA005 did not impair the viability of Tconv, while it inhibited the proliferation of Tmem and T_N_ at concentrations of 5 and 10 µM (Figure S1A–C).

### 2.2 Generation of Functional Suppressive Tregs by IFA005

**FIGURE 2:**
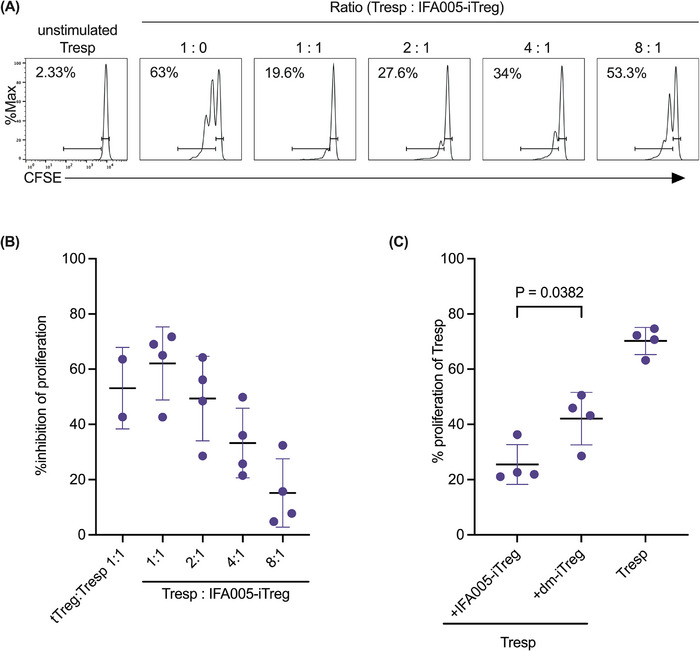
Suppression capacity of expanded IFA005‐iTregs or dm‐iTregs. IFA005‐iTregs or dm‐iTregs were co‐cultured with freshly isolated autologous CFSE^+^CD4^+^ responder T cells (Tresp) in the presence of Treg suppression inspector for 72 h. The proliferation of Tresp was analyzed by CFSE dilution. All conditions were duplicated or triplicated in individual experiments. The dilution of CFSE was read by flow cytometry. Data were analyzed by one‐way ANOVA. (A) Representative flow cytometry histograms show the proliferation of responder T cells under different conditions, and the proliferation was assessed by proliferation dye CFSE. (B) Percentage suppression of Tresp proliferation in the presence of tTreg (*n* = 2) at a 1:1 ratio or of IFA005‐iTreg at indicated cell ratios (*n* = 4). (C) The proliferation of Tresp in the presence of IFA005‐iTregs or dm‐iTregs at a 1:1 cell ratio (*n* = 4). In (B, C), the mean of 3 or 4 individual experiments ± SD is shown. Each dot represents the mean of duplicates or triplicates performed in an individual experiment. Paired *t*‐test was applied for statistics in (C).

Memory CD4^+^T cells are a critical component of the adaptive immune system and drivers of autoimmunity and chronic inflammation. Therefore, we explored Tregs converted from human primary memory CD4^+^T cells for their immunosuppressive effects. IFA005‐converted iTregs showed high surface levels of cytotoxic T‐lymphocyte‐associated antigen 4 (CTLA4) [25], TIGIT [26], CD39 [27], and Inducible T‐cell costimulatory ICOS [28], all of which are known to promote the suppressive activity of Tregs. Specifically, IFA005 significantly upregulated the expression of ICOS (*p* = 0.007) and CD39 (*p* = 0.001) among Tmem. CTLA4 and TIGIT were elevated across all experiments, but the increases did not reach statistical significance (Figure 1F). We further investigated the suppressive function of IFA005‐iTregs through a Treg suppression assay. IFA005‐iTregs showed a robust inhibition of Tresp proliferation at a ratio of 1:1 (62 % ± SD 13), comparable to tTregs (53% ± SD 15) (Figure 2A,B). This inhibitory effect was observed across different ratios of Tresp: IFA005‐iTregs tested. In particular, IFA005‐iTregs showed significantly higher suppressive activity than dm‐iTregs (Figure 2C). Furthermore, Foxp3 expression was markedly higher in CD25^high^ IFA005‐iTregs compared with CD25^low^ IFA005‐treated Tmem (Figure S2B). Notably, the suppressive function of IFA005‐iTregs was preserved even after a 3‐day culture in the absence of IFA005, highlighting the stability of the induced Treg phenotype (Figure S3). Thus, IFA005 treatment effectively promoted an immunomodulatory memory Tconv phenotype with features of tTregs.

### 2.3 IFA005 Downregulates Proinflammatory Cytokine Secretion in Memory CD4^+^T Cells and IFA005‐iTregs

**FIGURE 3:**
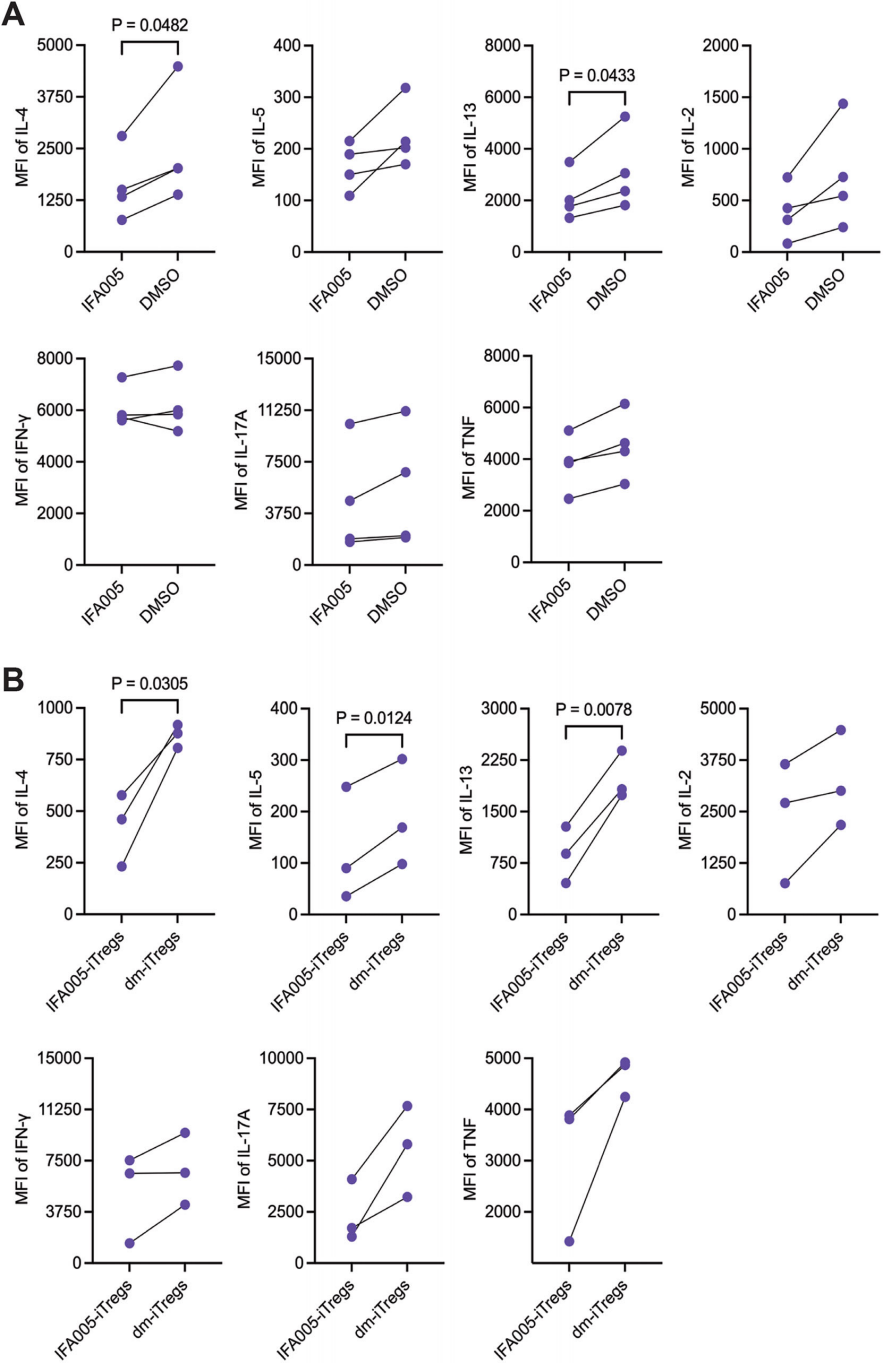
Cytokine profile of memory CD4^+^T cells and IFA005‐iTregs. (A) Tmem were stimulated with plate‐bound anti‐CD3/CD28 mAb for 3 days in the presence or absence of IFA005 (1 µM). Supernatants were collected and cytokine levels were measured using Luminex xMAP technology (*n* = 4). (B) IFA005‐iTregs and dm‐iTregs were stimulated with PMA/Iono for 5 h, supernatants were collected and measured (*n* = 3). The median fluorescence intensity (MFI) as readout of the Luminex instrument is shown. All data were presented as mean ± SD; a paired *t*‐test was applied for statistics. All data points in graphs represent individual experiments of individual donors.

Cytokines are essential signaling molecules that coordinate local and systemic immune cell functions, modulate immune responses, and enhance immune cell differentiation. To delineate the cytokine signature in IFA005‐treated cells, we assessed cytokine levels secreted by IFA005‐iTregs and within memory CD4^+^T cells in the presence of IFA005. In the presence of IFA005, memory CD4^+^T cells secreted significantly less Th2 cytokines including IL‐4 (*p* = 0.05) and IL‐13 (*p* = 0.04) (Figure 3A). At the same time, the production of IL‐2, IFN‐γ, TNF, and IL‐17 was reduced, although not reaching statistical significance (Figure 3A). IFA005‐iTregs showed a statistically significant reduction in the secretion of IL‐4 (*p* = 0.03), IL‐5 (*p* = 0.01), and IL‐13 (*p* = 0.008) (Figure 3B). The levels of IL‐2 and IL‐17 were decreased, but without reaching statistical significance. No differences in IFN‐γ and TNF levels were observed (Figure 3B). Furthermore, no differences in IL‐10 secretion between IFA005‐treated and control, or between IFA005‐iTregs and dm‐iTregs, were found (Figure S4). Overall, our findings show that treatment with IFA005 led to similar cytokine secretion patterns in both the total pool of IFA005‐treated memory CD4^+^T cells and IFA005‐iTregs. Notably, we observed a significant decrease in the production of proinflammatory Th2‐type cytokines in IFA005‐treated memory CD4^+^T cells and IFA005‐iTregs, while no upregulation of proinflammatory cytokines was detected.

### 2.4 IFA005 iTregs Are Epigenetically Different from tTregs

**FIGURE 4:**
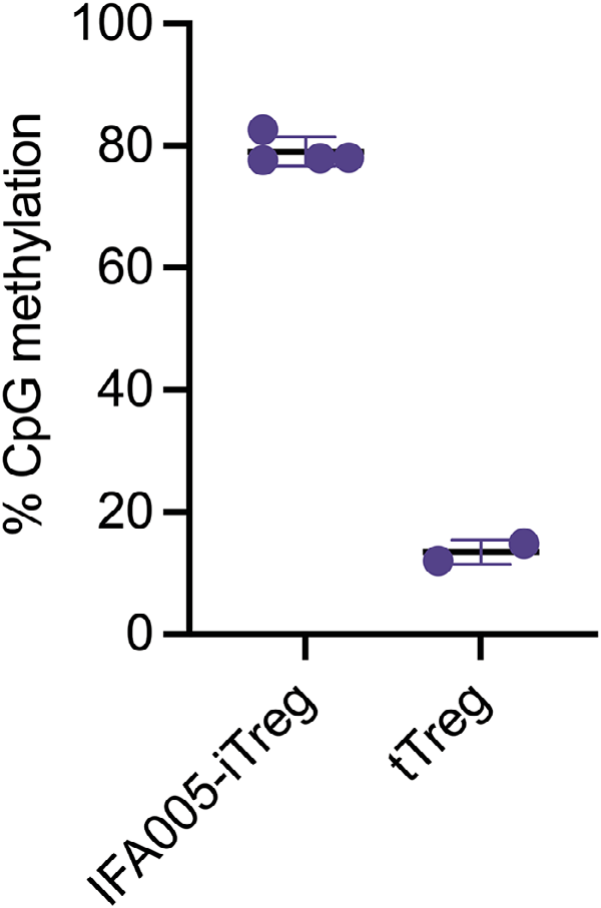
IFA005‐iTregs are epigenetically different from tTregs. IFA005‐iTregs were generated from Tmem (*n* = 3), and tTreg (*n* = 2) were sorted ex vivo from autologous PBMC. The methylation status of the FOXP3‐TSDR was analyzed using bisulfite amplicon sequencing and is displayed as the mean methylation degree overall included CpG sites. Data were presented as mean ± SD; data points in the graph represent individual donors.

Foxp3 expression plays a critical role in Treg development, but its presence alone is not sufficient to establish a Treg cell population. Epigenetic modifications, particularly hypomethylation of CpG dinucleotides within the evolutionarily conserved Treg‐specific demethylated region (TSDR) in the FOXP3 gene locus, are essential for the generation of a durable suppressor cell lineage [29, 30]. High demethylation of the TSDR, which is linked to stable Foxp3 expression, is a critical feature of tTreg. Tconv and TGF‐β‐induced iTreg exhibit TSDR hypermethylation [31]. To investigate the influence of IFA005 treatment on the methylation status of Tconv, we analyzed all 15 CpG sites within the TSDR by bisulfite amplicon sequencing. The TSDR within IFA005‐iTregs was hypermethylated compared with tTregs (Figure 4) and was not altered from dm‐iTregs (Figure S5). Our results indicate that despite its suppressive capabilities, IFA005‐iTregs may not undergo conversion to an epigenetically imprinted phenotype corresponding to that of tTregs.

### 2.5 IFA005 Treatment Upregulates NKD2 in Memory CD4^+^T Cells

**FIGURE 5:**
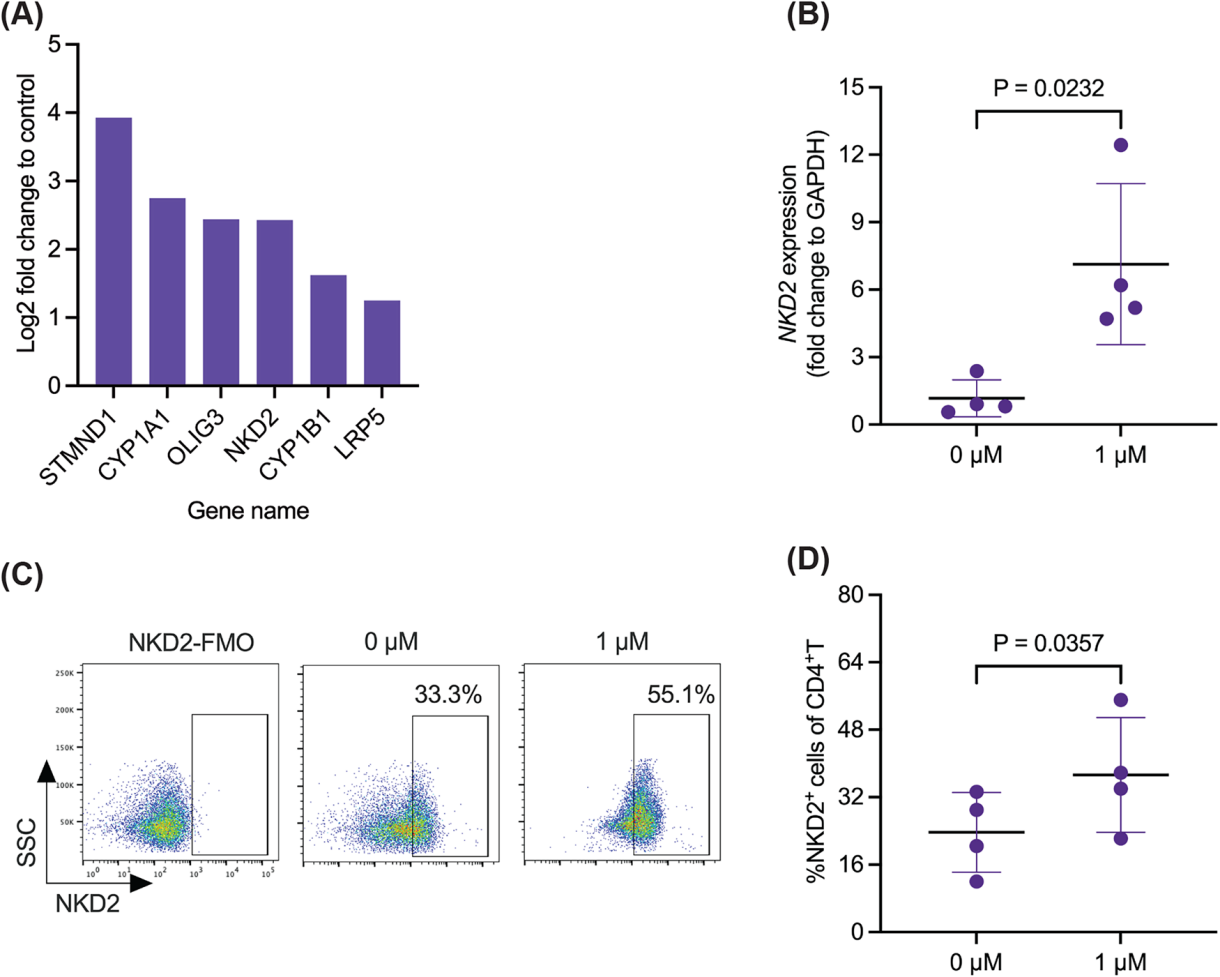
NKD2 is upregulated in IFA005‐treated memory CD4^+^T cells. Tmem were stimulated with plate‐bound anti‐CD3/CD28 mAb in the presence or absence of 1 µM of IFA005 for 3 days. (A) Total RNA was isolated, followed by RNA sequencing. Log2 fold change of expression of 6 DEGs in IFA005‐treated Tmem is shown (*n* = 3). (B) RT‐qPCR was performed, and the fold change expression of *NKD2* with respect to *GAPDH* in individual experiments is shown (*n* = 4). Representative flow cytometry dot plots (C) and scatter diagrams (D) show the expression of NKD2 (*n* = 4). All data were presented as mean ± SD; paired *t*‐test was applied for statistics in (B–D); *p*‐value is indicated. Data points in (B–D) represent individual experiments of individual donors.

To further investigate the mechanism of action of IFA005 on memory CD4^+^T cells during conversion into iTregs, we analyzed the transcriptional profile of IFA005‐treated memory CD4^+^T cells by RNA‐sequencing. We identified 30 differentially expressed genes (Figure S6) and six significantly upregulated genes (Figure 5A), including naked cuticle homolog 2 (NKD2, log2 fold upregulation = 2.43). NKD2 is an adapter protein and endogenous inhibitor of Wnt‐β‐catenin signaling [32] and has been described to reduce breast cancer cell proliferation [33] and tumor and metastasis growth in osteosarcoma patients [34]. Notably, Wnt‐β‐catenin signaling has previously been documented to suppress Foxp3 expression and Treg suppressive activity via the transcription factor T cell factor 1 (TCF) [35, 36]. Since the impact of NKD2 expression on Wnt‐β‐catenin signaling in the context of generation of regulatory T cells has not been reported, we prioritized to delineate the interactions among IFA005, NKD2, and the Wnt‐β‐catenin pathway. Using quantitative real‐time PCR (RT‐qPCR), we observed a substantial upregulation of *NKD2* in response to IFA005 (*p* = 0.02) (Figure 5B). Correspondingly, flow cytometry analysis revealed a significant augmentation in intracellular NKD2 protein levels at a concentration of 1 µM of IFA005 (*p* = 0.04) (Figure 5C). These findings underline the potent capacity of IFA005 to induce NKD2 expression in human memory CD4^+^T cells in vitro, which can be linked to increased Foxp3 levels and the induced suppressive phenotype of IFA005‐iTregs.

### 2.6 IFA005 Interferes With the Wnt‐β‐Catenin Pathway

**FIGURE 6:**
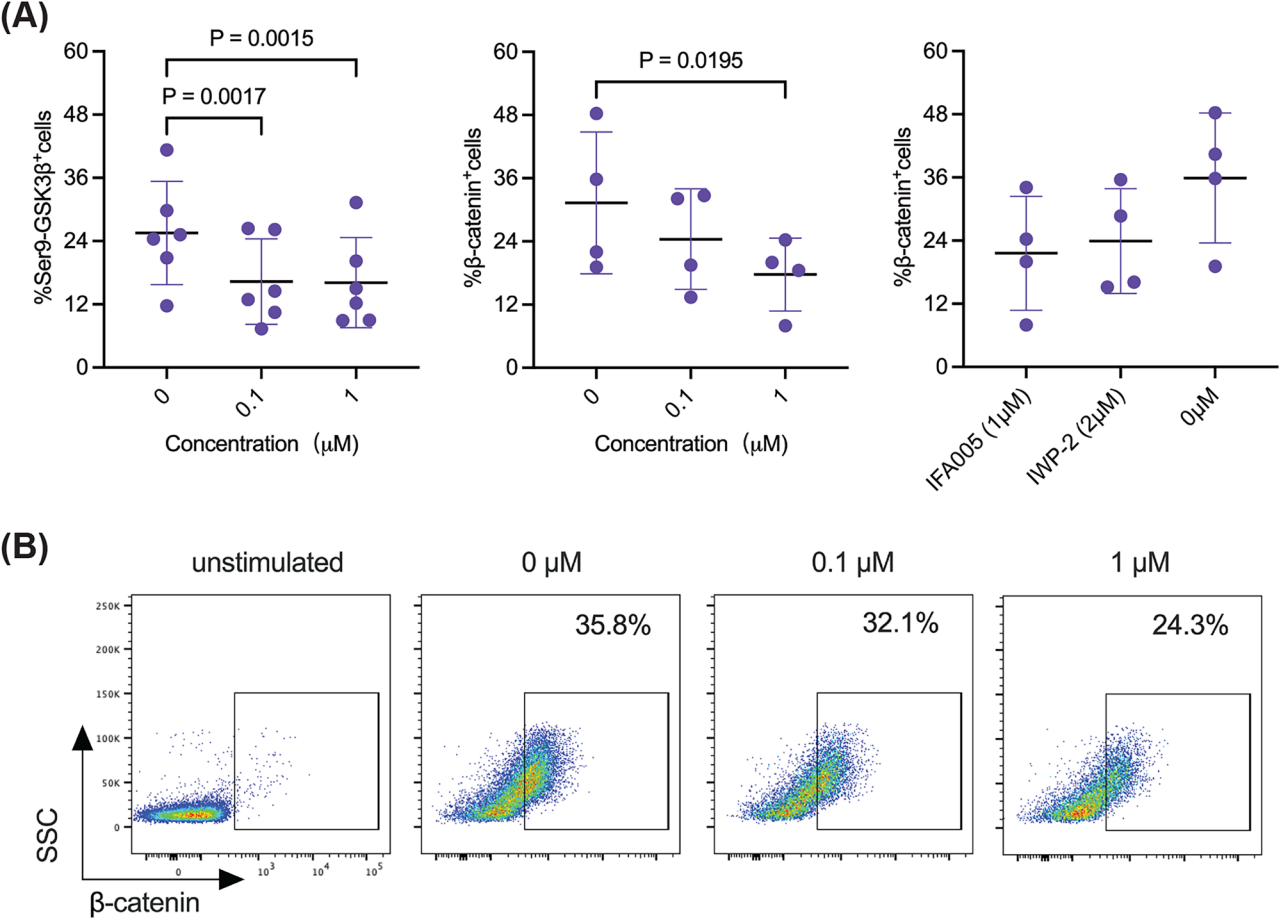
IFA005 regulates β‐catenin and Ser9‐GSK3β in memory CD4+T cells. Tmem were activated with plate‐bound anti‐CD3/CD28 mAb in the presence or absence of the indicated concentration of IFA005 or the inhibitor of Wnt signaling IWP2 for 3 days. (A) The expression of Ser9‐GSK3β in Tmem (*n* = 6). (B) The frequency of β‐catenin‐expressing T cells (*n* = 4). IWP2, the inhibitor of the Wnt signaling pathway, was used as a positive control for β‐catenin inhibition. (D) Representative flow plots show the expression of β‐catenin in live Tmem. All data are presented as mean ± SD. *p*‐values are displayed, and one‐way ANOVA was used to calculate statistical differences. Data points in (A–C) represent individual experiments of individual donors. Created with BioRender.com.

Canonical Wnt signaling induces the inactivation of glycogen synthase kinase‐3β (GSK3β) [37], which is associated with degradation of β‐catenin and TCF1 activity, which opposes Foxp3 expression [35]. GSK3β activity is mainly regulated by phosphorylation at Ser9 [38], which can be directly linked to β‐catenin levels [39]. In contrast, NKD2 interrupts the transduction of Wnt signals via binding to disheveled [40], which promotes the degradation of β‐catenin and thereby reduces canonical Wnt‐β‐catenin signaling. Based on these previous observations and our own Foxp3 data (Figure 1), we hypothesized that increased expression of NKD2 upon IFA005 treatment leads to reduced phosphorylation of Ser9 of GSK3β, resulting in reduced β‐catenin levels, and ultimately, upregulation of Foxp3. To test this hypothesis, we measured the phosphorylation status of GSK3β at Ser9 as well as levels of β‐catenin in memory CD4^+^T cells in the presence of IFA005. IFA005 treatment elicited a dose‐dependent and significant decrease in the phosphorylation level of Ser‐9 (Figure 6A), indicating increased activation of GSK3β. Notably, a substantial reduction in Ser‐9 phosphorylation was already evident with 100 nM IFA005, suggesting potent interaction with GSK3β. Consistent with this observation, a dose‐dependent decline in β‐catenin levels was noted upon IFA005 treatment (Figure 6B,D). Comparative analysis of β‐catenin levels following IFA005 treatment and treatment with the Wnt inhibitor IWP2 [41] demonstrated similar activity in downregulating β‐catenin levels in memory CD4^+^T cells (Figure 6C). However, generated IWP2‐iTregs failed to suppress proliferation of Tresp as compared with IFA005‐iTregs (Figure S7), suggesting that IWP2 lacks the capacity to induce functional iTregs. These results indicate that NKD2‐mediated downregulation of canonical Wnt/β‐catenin signaling is a key driver of the conversion of T conv into a suppressive CD4^+^iTreg cell phenotype.

## 3 Discussion

In this study, we identified a previously uncharacterized quinoxaline derivative, 9‐methoxy‐6‐methylindolo[3,2‐b] quinoxaline (IFA005), that promotes a phenotypic and functional conversion of Tconv into Treg‐like cells independent of TGF‐β and APCs and without exhibiting toxicity. Thus, IFA005 has a greater efficacy compared with immunomodulatory agents such as retinoic acid [42], rapamycin [43], butyrate [44], and CTLA‐4 peptide [45], which are TGF‐β‐dependent and do not promote conversion of human memory T cells. IFA005‐iTreg cells were phenotypically similar to tTreg cells since levels of Foxp3, CTLA4, TIGIT, CD39, and ICOS were concomitantly increased. Constitutive expression or upregulation of these proteins was previously associated with Treg cell‐mediated suppression of effector cells [26, 46, 47, 48]. Indeed, IFA005‐iTregs strongly suppressed the proliferation of autologous CD4^+^CD25^−^Tconv cells, demonstrating that IFA005‐iTregs are also functionally similar to tTregs. In addition, secretion of the Th2 cytokines IL‐4, IL‐5, and IL‐13 was strongly reduced in IFA005‐iTregs and also total IFA005‐treated memory CD4^+^T cells, while proinflammatory cytokines (i.e., IL‐2, TNF, IFN‐γ, IL‐17) were moderately downregulated. Thus, IFA005 effectively mediates the conversion of effector T cell phenotype toward a regulatory, anti‐inflammatory state.

Based on our epigenetic analyses, it appears that this conversion may not be fully imprinted since IFA005‐iTregs lacked a demethylated TSDR, a hallmark of tTreg cells necessary for manifesting the Treg phenotypic and functional stability [29, 31]. Nevertheless, IFA005‐iTregs may provide benefits in pathological conditions, specifically allergies, which are characterized by a shortage of antigen‐specific Treg cells [49]. In these scenarios, modulation of immune effector mechanisms may be sufficient to hinder clonal expansion of effector cells, alleviate inflammation, and promote immune homeostasis over time. Importantly, there is a significant medical need for enhancing the repertoire of therapeutic interventions that target type 2 inflammatory conditions, such as (auto)allergies, asthma, and atopic dermatitis [50, 51]. The substantial downregulation of IL‐4, IL‐5, and IL‐13 in IFA005‐iTregs and IFA005‐treated memory Tconv, together with the transient establishment of a Treg phenotype, suggests this as a promising treatment option for type 2 inflammation.

Several mechanisms may be required to effectively convert Tconv into functional Treg cells, as the targeted demethylation of the Foxp3 TSDR alone does not appear to be sufficient to induce functional Tregs and upregulation of suppressive receptors [52]. Here we show that one of these mechanisms is the blocking of Wnt‐β‐catenin by upregulation of NKD2, a negative regulator of the canonical Wnt‐β‐catenin signaling, through IFA005. The role of NKD2 and its inhibitory impact on the WNT signaling pathway has previously been documented in the context of suppressing tumor growth and metastasis in osteosarcoma [34], colorectal carcinoma [53], and acute myeloid leukemia [54]. Here, we provide evidence that an increased level of NKD2 supports the induction of Treg functions in Tconv, which has not been described so far. We observed that upregulation of NKD2 at gene and protein levels in Tconv upon treatment with IFA005 was associated with reduced phosphorylation at Ser9 of GSK3β, which promoted degradation of β‐catenin.

Several studies highlight the involvement of Wnt‐β‐catenin in cancer, inflammation, T cell biology, and, importantly, in shaping T cell phenotypes. Increased β‐catenin expression was associated with a conversion of Treg cells into proinflammatory cells, showing upregulation of retinoic acid–related orphan receptor‐γt (RORγt), IL‐17 [55], and induction of proinflammatory properties in Treg and T cells [56]. The Wnt‐β‐catenin signaling pathway induces TCF1, which competes with Foxp3, leading to the downregulation of the suppressive capacities observed in tTregs and TGF‐β‐induced Tregs [35]. Conversely, the inhibition of Wnt‐β‐catenin facilitates Foxp3‐mediated inhibition of target genes, including IL‐2 [57]. Notably, treatment with Fingolimod (FTY720) in human naïve T cells reduced the phosphorylation of GSK3β, resulting in decreased levels of β‐catenin and subsequent reductions in the secretion of proinflammatory cytokines such as IFN‐γ, IL‐17, GM‐CSF, TNF, and granzyme B [58].

In a previous study, the successful conversion of effector Tconv into functional iTreg was achieved through the inhibition of the mediator complex kinase cyclin‐dependent kinases CDK8/19 using a small molecule compound [21, 22]. Notably, CDK8 has been identified as an oncogene [59] and an enhancer of Wnt signaling, with its elevated level and activity promoting colon cancer cell proliferation [59, 60]. Furthermore, the activity of CDK8 has been directly associated with β‐catenin levels and the expression of target genes, underscoring the pivotal role of Wnt‐β‐catenin in shaping T cell phenotypes. The identification of a quinoxaline derivative in this study highlights its widespread pharmacological properties and versatility as a therapeutic molecule. Quinoxalines are widely used in medical chemistry and possess potent immunomodulatory properties in various immune cells, including T cells [61], mast cells [62], and eosinophils [63]. Quinoxaline derivatives have been recently marketed for the treatment of viral infections, cancers, or are in clinical trials, for example, rheumatoid arthritis [64]. Based on the advantageous physico‐chemical properties of these compounds, IFA005 is an ideal structure for further medicinal chemistry development of similar molecules with even more targeted properties.

In summary, our study establishes 9‐methoxy‐6‐methylindolo[3,2‐b] quinoxaline as a potent inducer of functional Foxp3^+^ Treg cells derived from human memory CD4^+^T cells. This compound not only effectively suppresses IL‐4, IL‐5, and IL‐13 but also disrupts Wnt‐β‐catenin signaling and de‐stabilizes β‐catenin in vitro, suggesting its potential efficacy in addressing Th2‐driven conditions such as chronic allergic inflammation. Furthermore, our findings emphasize the pivotal role of NKD2 in the regulation of Wnt‐β‐catenin signaling, promoting the Treg cell phenotype and proposing innovative therapeutic avenues for restoring immune tolerance.

In conclusion, the targeting of WNT‐β‐catenin signaling emerges as a promising strategy for immunomodulation in chronic inflammatory diseases.

### 3.1 Data Limitations and Perspectives

This study focuses only on the effects of IFA005 in total human memory CD4⁺ T cells under noninflammatory conditions. Future studies are needed to evaluate the functional impact of IFA005 in inflammatory contexts that more closely reflect autoimmune or allergic disease settings. In particular, dissecting its ability to convert specific effector T cell subsets, such as Th1, Th2, and Th17 cells, into regulatory T cells will be crucial for assessing its therapeutic potential. Such investigations may also support the development of tailored or personalized immunomodulatory approaches. Moreover, while our findings highlight a link between NKD2 and Foxp3 expression, the current study does not provide direct mechanistic evidence that NKD2 is solely required for IFA005‐induced Treg conversion. Future experiments involving NKD2 knockdown or knockout are necessary to determine whether NKD2 plays a causal role in this process or acts in combination with other signaling pathways. These studies will be critical to fully elucidate the mechanism of action of IFA005 and its utility as an immunomodulatory agent.

## 4 Materials and Methods

### 4.1 Mice

Foxp3‐eGFP reporter mice (DEREGxDo11.10) [65] were bred at the animal facility of Charité Universitätsmedizin Berlin and maintained in accordance with institutional and federal animal welfare guidelines.

### 4.2 Blood Samples and Cell Isolation

Peripheral blood mononuclear cells (PBMCs) were isolated by Ficoll‐Paque (GE Healthcare Life Sciences). For PBMC cryopreservation, 2 × 10^7 ^PBMC were gently resuspended in cold freezing medium consisting of 10% dimethyl sulfoxide (DMSO) and 90% fetal bovine serum (FBS), then transferred into cryovials (Sarstedt) and stored at −80°C using a freezing container (Mr. Frosty) filled with isopropanol. Human naïve CD4^+^T cells and memory CD4^+^T cells were enriched with Naïve CD4^+^T cell isolation Kit II, human and Memory CD4^+^T cell isolation kit, human (Miltenyi Biotec), respectively, from PBMCs. CD4^+^CD25^hi^CD127^lo^ regulatory T cells (tTregs) were sorted from pre‐enriched CD4^+^T cells via FACSArias II (BD Biosciences). The purity of enriched CD4^+^, naïve CD4^+^T, memory CD4^+^T, and tTregs is above 90%.

### 4.3 High‐Throughput Screening

Primary cells from spleen and lymph nodes of Foxp3‐eGFP reporter mice were mixed and depleted of CD8+ T cells by magnetic separation (Miltenyi Biotec). 3 × 10^5^ cells per well were seeded in 384‐well plates and cultured in the presence of individual compounds from a library consisting of 41,184 small molecules, including drugs from Selleck, world drug index (WDI) derived molecules [66], the library of pharmacologically active compounds (LOPAC, Sigma Aldrich), and noncommercial compounds from academia (Leibniz Forschungsinstitut für molekulare Pharmakologie, Berlin). Ovalbumin (1 µg/mL, in‐house) and IL‐2 (10 ng/mL, R&D Systems) were added to the cell suspension for stimulation. The positive control additionally contained TGF‐β (5 ng/mL, R&D Systems) as an inducer of Foxp3 expression. Incubation was performed at 37°C for 72 h, and the cells were stained with antibodies against CD4 (GK 1.5; in‐house) and PI (1 µg/mL, Merck), measured using the HyperCyt autosampler (Intellicyt) coupled to a flow cytometer (Accuri, BD). A threshold of 3σ according to untreated controls (*n* = 16 on each assay plate) was set to identify Foxp3‐inducing compounds. Identified hit compounds were purchased from ChemDiv. IFA005 was synthesized as described previously by Shulga et al. [67].

### 4.4 T Cell Stimulation and iTregs Induction

Tconv were stimulated with plate‐bound anti‐CD3/CD28 (Biolegend) mAb in X‐VIVO 15 (Lonza) + Penicillin‐Streptomycin solution (Cytiva) + 25 IU/ml IL‐2 (Miltenyi Biotec). For T‐cell stimulation, the culture plates were coated with 1 µg/ml anti‐CD3/CD28 mAb for more than 2 h at 37°C before seeding the cells. For inducing iTregs, memory CD4^+^T cells were incubated for 3 days in the presence of 1 µM of IFA005 in serum‐free X‐VIVO 15. CD25^high^CD4^+^T cells sorted from the whole cell pool were defined as IFA005‐iTregs. Control cells treated with mock (DMSO) were defined as dm‐iTregs. The sorting strategy and corresponding Foxp3 level are shown in Figure S2A,B.

### 4.5 Flow Cytometry

MACSQuantX analyzer (Miltenyi Biotech) and Sony ID7000 spectral cell analyzer (Sony Biotechnology) were used for acquisition. The data were analyzed using the FlowJo software 10.0 (BD). Surface marker staining was performed with the following antibodies and fluorescent dyes: anti‐CD4 BV510 and anti‐TIGIT BV421 (BioLegend); anti‐CD4 percp, anti‐CD45RA PE‐Vio 770, anti‐CD45RO Vio‐Green, anti‐CD127 APC, and anti‐CD25 PE (Miltenyi Biotec); anti‐CD39 BV510 and anti‐ICOS BB515 (BD Bioscience). Cell proliferation was done with cell proliferation dye eFluor 670 (eF670) or CFSE, and cell viability was assessed by fixable viability dye eFluor 780 (eF780). Foxp3 and CTLA‐4 intracellular staining were performed with anti‐CTLA‐4 APC (Miltenyi Biotec) and anti‐Foxp3 PE (Thermo Fisher) using Foxp3/transcription factor staining buffer set (Thermo Fisher) according to the manufacturer's protocol. For β‐catenin, Ser9‐GSK3β, and NKD2 staining, cells were fixed with 2% formaldehyde, permeabilized with 90% methanol, then labeled with anti‐β‐catenin AF647, anti‐Ser9‐GSK3β AF647, or Naked2 Rabbit mAb separately (Cell Signaling Technology). To determine NKD2, anti‐rabbit IgG AF647 (Cell Signaling Technology) was used.

### 4.6 iTreg Suppression Assay

Autologous CD4^+^CD25^−^T cells were used as responder T cells (Tresp) and were sorted from frozen PBMCs. Tresp was labeled with 5 µM of CFSE right prior to co‐culture. Freshly sorted tTregs were used as a positive control. Tresp was plated with gradient iTregs or tTregs in a 96‐well round‐bottom plate (2 × 10^4^ to 4 × 10^4^ cells per well) in the presence or absence of Treg suppression inspector (Miltenyi Biotec) for 72 h; the ratio of bead to cell was 1:1. All conditions were duplicated or triplicated. To assess the stability of IFA005‐iTregs, sorted IFA005‐iTregs or dm‐iTregs were cultured for 3 days in medium (X‐VIVO 15 supplemented with penicillin/streptomycin, 10% FBS, and 100 IU/mL IL‐2) without IFA005, then co‐cultured with freshly isolated autologous CFSE‐labeled CD4⁺CD25^−^ responder T cells (Tresp) in the presence of Treg Suppression Inspector for 72 h. Proliferation was assessed by CFSE dilution, and the percentage suppression of proliferation was calculated by relating the percentage of proliferating Tresp in the presence and absence of IFA005‐iTregs, respectively.

### 4.7 TSDR Methylation via Bisulfite Amplicon Sequencing

IFA005‐iTregs and tTregs were harvested and stored in liquid nitrogen, and 250 ng of genomic DNA was bisulfite‐converted with EZ DNA Methylation Kit (Zymo Research). The TSDR was amplified by PCR using KAPA HiFi Hotstart Uracil+ ReadyMix (Roche) with primers containing partial Illumina adapter sequences. Amplicons were purified with AMPure XP beads (Beckman Coulter), and sizes were confirmed by Bioanalyzer (Agilent). Amplicons were submitted to Genewiz for next‐generation sequencing. After standard preprocessing of raw sequencing files, alignment and methylation calls of sequencing reads were performed using the software Bismark [68]. Data visualization and statistics were performed on R using ggpubr [69].

### 4.8 Cytokine Measurement

To assess the cytokines in the supernatants of memory CD4^+^T cells, Tmem were stimulated with plate‐bound anti‐CD3/CD28 mAb in the absence or presence of 1 µM of IFA005 for 3 days, then supernatants were collected. For analyzing cytokine levels in IFA005‐iTregs and dm‐iTregs, IFA005‐iTregs and dm‐iTregs were sorted as mentioned above, and then stimulated with 50 ng/mL phorbol 12‐myristate 13‐acetate (PMA, Sigma‐Aldrich) and 500 ng/mL ionomycin (Iono, Sigma‐Aldrich) for 5 h, and supernatants were collected. Cytokines were detected using a multiplex cytokine array (ProcartaPlex Human Cytokine Panel 1, Thermo Fisher Scientific), measured by the Luminex MAGPIX instrument. Median fluorescence intensity values of streptavidin‐phycoerythrin (PE) signals, which are proportional to the amount of bound analyte, were used for data analysis.

### 4.9 RNA‐sequencing

Human primary memory CD4^+^T cells were stimulated with or without IFA005 for 3 days. Total RNA was isolated with NucleoSpin RNA isolation kit (Macherey‐Nagel). RNA quality control, library preparation, sequencing, and data analysis were performed by Brooks Life Science Genewiz.

### 4.10 Reverse transcription‐quantitative PCR

Total RNA was isolated as mentioned in the RNA‐sequencing section. cDNA was synthesized with PrimeScriptTM RT reagent kit (Katara). Gene expression was monitored using real‐time primer pairs with SYBR Green detection (Applied Biosystems, Life Technology) and analyzed using SteOnePlus real‐time PCR machine (AB Biosystems, Life Technology). Relative fold change in mRNA expression compared with the control was calculated using the ΔΔC_t_ method [70]. All samples were normalized to GAPDH expression levels.

### 4.11 Statistics

Statistical analyses were performed with GraphPad Prism 10. One‐way ANOVA or *t*‐test was performed as indicated in the figure legends. Data are displayed if not indicated elsewhere as mean ± SD. *p*‐values ≤ 0.05 were considered significant. *p*‐values are displayed in figures only when *p* ≤ 0.05.

Further details on antibodies and reagents are listed in Table S1.

## Author Contributions

Jiajun He and Stefan Frischbutter designed the study. Stefan Frischbutter, Jiajun He, Kristy Ou, and Jérôme Paul performed the experiments. Jiajun He, Kristy Ou, and Stefan Frischbutter analysed data. Stefan Frischbutter, Jérôme Paul, Michael‐Schmueck‐Henneresse, Julia K. Polansky, Edgar Specker, Marc Nazare, Jens Peter von Kries, Marc Nazare, and Alf Hamann interpreted data. Jiajun He and Stefan Frischbutter wrote the manuscript. All authors critically revised and finally approved the manuscript.

## Ethics Approval Statement

This study was approved by the Institutional Review Board of Charité (EA1/116/13; EA1/292/14; EA2/286/21), and informed consent was obtained from all subjects. Buffy coats were obtained from Deutsches Rotes Kreuz (DRK), or fresh peripheral blood samples were obtained from healthy volunteers.

## Conflicts of Interest

The authors declare no conflicts of interest.

## Peer Review

The peer review history for this article is available at https://publons.com/publon/10.1002/eji.70018.

## Supporting information




**Supporting file 1**: eji70018‐sup‐0001‐SuppMat.pdf

## Data Availability

The raw data (fastq files) from the RNA‐seq are available from the corresponding author upon reasonable request. All data needed to evaluate the conclusions in the paper are present in the paper or the Supporting Information Materials.

## References

[1] F. W. Miller , “The Increasing Prevalence of Autoimmunity and Autoimmune Diseases: An Urgent Call to Action for Improved Understanding, Diagnosis, Treatment, and Prevention,” Current Opinion in Immunology 80 (2023): 102266. 36446151 10.1016/j.coi.2022.102266 PMC9918670

[2] H. Groux , A. O'Garra , M. Bigler , M. Rouleau , S. Antonenko , J. E. de Vries , et al., “A CD4+ T‐Cell Subset Inhibits Antigen‐specific T‐Cell Responses and Prevents Colitis,” Nature 389, no. 6652 (1997): 737–742. 9338786 10.1038/39614

[3] P. A. Taylor , R. J. Noelle , and B. R. Blazar , “CD4(+)CD25(+) Immune Regulatory Cells Are Required for Induction of Tolerance to Alloantigen via Costimulatory Blockade,” Journal of Experimental Medicine 193, no. 11 (2001): 1311–1318. 11390438 10.1084/jem.193.11.1311 PMC2193378

[4] F. Van Wijk , E. J. M. Wehrens , S. Nierkens , et al., “CD4+CD25+ T Cells Regulate the Intensity of Hypersensitivity Responses to Peanut, but Are Not Decisive in the Induction of Oral Sensitization,” Clinical & Experimental Allergy 37, no. 4 (2007): 572–581. 17430355 10.1111/j.1365-2222.2007.02681.x

[5] S. Sakaguchi , N. Mikami , J. B. Wing , A. Tanaka , K. Ichiyama , and N. Ohkura , “Regulatory T Cells and Human Disease,” Annual Review of Immunology 38 (2020): 541–566. 10.1146/annurev-immunol-042718-041717 32017635

[6] J. D. Fontenot , M. A. Gavin , and A. Y. Rudensky , “Foxp3 programs the Development and Function of CD4+CD25+ Regulatory T Cells,” Nature Immunology 4, no. 4 (2003): 330–336. 12612578 10.1038/ni904

[7] S. Hori , T. Nomura , and S. Sakaguchi , “Control of Regulatory T Cell Development by the Transcription Factor Foxp3,” Science 299, no. 5609 (2003): 1057–1061. 12522256 10.1126/science.1079490

[8] A. K. Abbas , C. Benoist , J. A. Bluestone , et al., “Regulatory T Cells: Recommendations to Simplify the Nomenclature,” Nature Immunology 14, no. 4 (2013): 307–308. 23507634 10.1038/ni.2554

[9] L. M. R. Ferreira , Y. D. Muller , J. A. Bluestone , and Q. Tang , “Next‐Generation Regulatory T Cell Therapy,” Nat Rev Drug Discovery 18, no. 10 (2019): 749–769. 31541224 10.1038/s41573-019-0041-4 PMC7773144

[10] J. A. Bluestone , J. H. Buckner , M. Fitch , et al., “Type 1 Diabetes Immunotherapy Using Polyclonal Regulatory T Cells,” Science Translational Medicine 7, no. 315 (2015): 315ra189. 10.1126/scitranslmed.aad4134 PMC4729454 26606968

[11] N. Marek‐Trzonkowska , M. Myśliwiec , A. Dobyszuk , et al., “Therapy of Type 1 Diabetes With CD4(+)CD25(high)CD127‐Regulatory T Cells Prolongs Survival of Pancreatic Islets—Results of One Year Follow‐up,” Clinical Immunology 153, no. 1 (2014): 23–30. 24704576 10.1016/j.clim.2014.03.016

[12] F. van Wijk , E. J. Wehrens , S. Nierkens , et al., “CD4+CD25+ T Cells Regulate the Intensity of Hypersensitivity Responses to Peanut, but Are Not Decisive in the Induction of Oral Sensitization,” Clinical and Experimental Allergy 37, no. 4 (2007): 572–581. 17430355 10.1111/j.1365-2222.2007.02681.x

[13] H. Yamashita , K. Takahashi , H. Tanaka , H. Nagai , and N. Inagaki , “Overcoming Food Allergy Through Acquired Tolerance Conferred by Transfer of Tregs in a Murine Model,” Allergy 67, no. 2 (2012): 201–209. 22050332 10.1111/j.1398-9995.2011.02742.x

[14] J. Kearley , J. E. Barker , D. S. Robinson , and C. M. Lloyd , “Resolution of Airway Inflammation and Hyperreactivity After in Vivo Transfer of CD4+CD25+ Regulatory T Cells Is Interleukin 10 Dependent,” Journal of Experimental Medicine 202, no. 11 (2005): 1539–1547. 16314435 10.1084/jem.20051166 PMC1350743

[15] J. H. Esensten , Y. D. Muller , J. A. Bluestone , and Q. Tang , “Regulatory T‐Cell Therapy for Autoimmune and Autoinflammatory Diseases: The next Frontier,” Journal of Allergy and Clinical Immunology 142, no. 6 (2018): 1710–1718. 30367909 10.1016/j.jaci.2018.10.015

[16] P. Zhou , “Emerging Mechanisms and Applications of Low‐dose IL‐2 Therapy in Autoimmunity,” Cytokine & Growth Factor Reviews 67 (2022): 80–88. 35803833 10.1016/j.cytogfr.2022.06.003

[17] M. Rosenzwajg , R. Lorenzon , P. Cacoub , et al., “Immunological and Clinical Effects of Low‐dose Interleukin‐2 Across 11 Autoimmune Diseases in a Single, Open Clinical Trial,” Annals of the Rheumatic Diseases 78, no. 2 (2019): 209–217. 30472651 10.1136/annrheumdis-2018-214229

[18] H. Torrey , W. M. Kühtreiber , Y. Okubo , et al., “A Novel TNFR2 Agonist Antibody Expands Highly Potent Regulatory T Cells,” Science signaling 13, no. 661 (2020): eaba9600. 33293464 10.1126/scisignal.aba9600

[19] X. Sun , Y. Xiao , Z. Zeng , et al., “All‐Trans Retinoic Acid Induces CD4+CD25+FOXP3+ Regulatory T Cells by Increasing FOXP3 Demethylation in Systemic Sclerosis CD4+ T Cells,” Journal of Immunology Research 2018 (2018): 8658156. 29854846 10.1155/2018/8658156 PMC5952589

[20] Y. Furusawa , Y. Obata , S. Fukuda , et al., “Commensal Microbe‐derived Butyrate Induces the Differentiation of Colonic Regulatory T Cells,” Nature 504, no. 7480 (2013): 446–450. 24226770 10.1038/nature12721

[21] M. Akamatsu , N. Mikami , N. Ohkura , et al., “Conversion of Antigen‐Specific Effector/Memory T Cells Into Foxp3‐expressing T(reg) Cells by Inhibition of CDK8/19,” Science Immunology 4, no. 40 (2019): eaaw2707. 31653719 10.1126/sciimmunol.aaw2707

[22] Z. Guo , G. Wang , Y. Lv , Y. Y. Wan , and J. Zheng , “Inhibition of Cdk8/Cdk19 Activity Promotes Treg Cell Differentiation and Suppresses Autoimmune Diseases,” Frontiers in Immunology 10 (2019). 10.3389/fimmu.2019.01988 PMC6736578 31552016

[23] A. Cossarizza , H. D. Chang , A. Radbruch , et al., “Guidelines for the Use of Flow Cytometry and Cell Sorting in Immunological Studies (second edition),” European Journal of Immunology 49, no. 10 (2019): 1457–1973. 31633216 10.1002/eji.201970107 PMC7350392

[24] L. Xu , A. Kitani , and W. Strober , “Molecular Mechanisms Regulating TGF‐beta‐Induced Foxp3 Expression,” Mucosal Immunol 3, no. 3 (2010): 230–238. 20404810 10.1038/mi.2010.7 PMC3673708

[25] S. Read , V. Malmström , and F. Powrie , “Cytotoxic T Lymphocyte–Associated Antigen 4 Plays an Essential Role in the Function of Cd25+Cd4+ Regulatory Cells That Control Intestinal Inflammation,” Journal of Experimental Medicine 192, no. 2 (2000): 295–302. 10899916 10.1084/jem.192.2.295 PMC2193261

[26] N. Joller , E. Lozano , P. R. Burkett , et al., “Treg Cells Expressing the Coinhibitory Molecule TIGIT Selectively Inhibit Proinflammatory Th1 and Th17 Cell Responses,” Immunity 40, no. 4 (2014): 569–581. 24745333 10.1016/j.immuni.2014.02.012 PMC4070748

[27] J. M. Fletcher , R. Lonergan , L. Costelloe , et al., “CD39+Foxp3+ Regulatory T Cells Suppress Pathogenic Th17 Cells and Are Impaired in Multiple Sclerosis,” Journal of Immunology 183, no. 11 (2009): 7602–7610. 10.4049/jimmunol.0901881 19917691

[28] D. Y. Li and X. Z. Xiong , “ICOS(+) Tregs: A Functional Subset of Tregs in Immune Diseases,” Frontiers in Immunology 11 (2020): 2104. 32983168 10.3389/fimmu.2020.02104 PMC7485335

[29] J. K. Polansky , K. Kretschmer , J. Freyer , et al., “DNA Methylation Controls Foxp3 Gene Expression,” European Journal of Immunology 38, no. 6 (2008): 1654–1663. 18493985 10.1002/eji.200838105

[30] S. Floess , J. Freyer , C. Siewert , et al., “Epigenetic Control of the foxp3 Locus in Regulatory T Cells,” PLoS Biology 5, no. 2 (2007): e38. 17298177 10.1371/journal.pbio.0050038 PMC1783672

[31] N. Ohkura , M. Hamaguchi , H. Morikawa , et al., “T Cell Receptor Stimulation‐induced Epigenetic Changes and Foxp3 Expression Are Independent and Complementary Events Required for Treg Cell Development,” Immunity 37, no. 5 (2012): 785–799. 23123060 10.1016/j.immuni.2012.09.010

[32] T. Hu , C. Li , Z. Cao , et al., “Myristoylated Naked2 Antagonizes Wnt‐Beta‐Catenin Activity by Degrading Dishevelled‐1 at the Plasma Membrane,” Journal of Biological Chemistry 285, no. 18 (2010): 13561–13568. 20177058 10.1074/jbc.M109.075945 PMC2859517

[33] Y. Dong , B. Cao , M. Zhang , et al., “Epigenetic Silencing of NKD2, a Major Component of Wnt Signaling, Promotes Breast Cancer Growth,” Oncotarget 6, no. 26 (2015): 22126–22138. 26124080 10.18632/oncotarget.4244 PMC4673151

[34] S. Zhao , L. Kurenbekova , Y. Gao , et al., “NKD2, a Negative Regulator of Wnt Signaling, Suppresses Tumor Growth and Metastasis in Osteosarcoma,” Oncogene 34, no. 39 (2015): 5069–5079. 25579177 10.1038/onc.2014.429 PMC4802362

[35] J. van Loosdregt , V. Fleskens , M. M. Tiemessen , et al., “Canonical Wnt Signaling Negatively Modulates Regulatory T Cell Function,” Immunity 39, no. 2 (2013): 298–310. 23954131 10.1016/j.immuni.2013.07.019

[36] M. Delacher , M. M. Barra , Y. Herzig , et al., “Quantitative Proteomics Identifies TCF1 as a Negative Regulator of Foxp3 Expression in Conventional T Cells,” Iscience 23, no. 5 (2020): 101127. 32422593 10.1016/j.isci.2020.101127 PMC7229326

[37] S. Piao , S. H. Lee , H. Kim , et al., “Direct Inhibition of GSK3beta by the Phosphorylated Cytoplasmic Domain of LRP6 in Wnt/Beta‐Catenin Signaling,” PLoS ONE 3, no. 12 (2008): e4046. 19107203 10.1371/journal.pone.0004046 PMC2603313

[38] V. Stambolic and J. R. Woodgett , “Mitogen Inactivation of Glycogen Synthase Kinase‐3 Beta in Intact Cells via Serine 9 Phosphorylation,” Biochemical Journal 303, no. Pt 3 (1994): 701–704. 7980435 10.1042/bj3030701 PMC1137602

[39] A. Kikuchi , S. Kishida , and H. Yamamoto , “Regulation of Wnt Signaling by Protein‐protein Interaction and Post‐Translational Modifications,” Experimental & Molecular Medicine 38, no. 1 (2006): 1–10. 16520547 10.1038/emm.2006.1

[40] D. Yan , J. B. Wallingford , T. Q. Sun , et al., “Cell Autonomous Regulation of Multiple Dishevelled‐Dependent Pathways by Mammalian Nkd,” PNAS 98, no. 7 (2001): 3802–3807. 11274398 10.1073/pnas.071041898 PMC31133

[41] B. Chen , M. E. Dodge , W. Tang , et al., “Small Molecule‐mediated Disruption of Wnt‐Dependent Signaling in Tissue Regeneration and Cancer,” Nature Chemical Biology 5, no. 2 (2009): 100–107. 19125156 10.1038/nchembio.137 PMC2628455

[42] M. J. Benson , K. Pino‐Lagos , M. Rosemblatt , and R. J. Noelle , “All‐trans Retinoic Acid Mediates Enhanced T Reg Cell Growth, Differentiation, and Gut Homing in the Face of High Levels of Co‐stimulation,” The Journal of Experimental Medicine 204, no. 8 (2007): 1765–1774. 17620363 10.1084/jem.20070719 PMC2118687

[43] M. Battaglia , A. Stabilini , B. Migliavacca , J. Horejs‐Hoeck , T. Kaupper , and M.‐G. Roncarolo , “Rapamycin Promotes Expansion of Functional CD4+CD25+FOXP3+Regulatory T Cells of both Healthy Subjects and Type 1 Diabetic Patients,” The Journal of Immunology 177, no. 12 (2006): 8338–8347. 17142730 10.4049/jimmunol.177.12.8338

[44] N. Arpaia , C. Campbell , X. Fan , et al., “Metabolites Produced by Commensal Bacteria Promote Peripheral Regulatory T‐Cell Generation,” Nature 504, no. 7480 (2013): 451–455. 24226773 10.1038/nature12726 PMC3869884

[45] G. R. Kim , W. J. Kim , S. Lim , et al., “In Vivo Induction of Regulatory T Cells via CTLA‐4 Signaling Peptide to Control Autoimmune Encephalomyelitis and Prevent Disease Relapse,” Adv Sci (Weinh) 8, no. 14 (2021): 2004973. 34306974 10.1002/advs.202004973 PMC8292875

[46] K. Wing , Y. Onishi , P. Prieto‐Martin , et al., “CTLA‐4 Control Over Foxp3+ Regulatory T Cell Function,” Science 322, no. 5899 (2008): 271–275. 18845758 10.1126/science.1160062

[47] M. Vocanson , A. Rozieres , A. Hennino , et al., “Inducible Costimulator (ICOS) Is a Marker for Highly Suppressive Antigen‐specific T Cells Sharing Features of TH17/TH1 and Regulatory T Cells,” Journal of Allergy and Clinical Immunology 126, no. 2 (2010): 280–289. 20624644 10.1016/j.jaci.2010.05.022

[48] J. Gu , X. Ni , X. Pan , et al., “Human CD39hi Regulatory T Cells Present Stronger Stability and Function Under Inflammatory Conditions,” Cellular & Molecular Immunology 14, no. 6 (2017): 521–528. 27374793 10.1038/cmi.2016.30 PMC5518817

[49] P. Bacher , F. Heinrich , U. Stervbo , et al., “Regulatory T Cell Specificity Directs Tolerance versus Allergy Against Aeroantigens in Humans,” Cell 167, no. 4 (2016): 1067–1078.e16. 27773482 10.1016/j.cell.2016.09.050

[50] P. Kolkhir , C. A. Akdis , M. Akdis , et al., “Type 2 Chronic Inflammatory Diseases: Targets, Therapies and Unmet Needs,” Nat Rev Drug Discovery 22, no. 9 (2023): 743–767. 37528191 10.1038/s41573-023-00750-1

[51] L. E. M. de Wijs , S. van Egmond , A. C. A. Devillers , T. Nijsten , D. Hijnen , and M. Lugtenberg , “Needs and Preferences of Patients Regarding Atopic Dermatitis Care in the Era of New Therapeutic Options: A Qualitative Study,” Archives of Dermatological Research 315, no. 1 (2023): 75–83. 35112162 10.1007/s00403-021-02321-z PMC8809237

[52] C. Kressler , G. Gasparoni , K. Nordström , et al., “Targeted De‐Methylation of the FOXP3‐TSDR Is Sufficient to Induce Physiological FOXP3 Expression but Not a Functional Treg Phenotype,” Frontiers in immunology 11 (2020): 609891. 33488615 10.3389/fimmu.2020.609891 PMC7817622

[53] X. J. Yang , C. Q. Huang , C. W. Peng , J. X. Hou , and J. Y. Liu , “Long Noncoding RNA HULC Promotes Colorectal Carcinoma Progression Through Epigenetically Repressing NKD2 Expression,” Gene 592, no. 1 (2016): 172–178. 27496341 10.1016/j.gene.2016.08.002

[54] X. X. Li , J. D. Zhou , T. J. Zhang , et al., “Epigenetic Dysregulation of NKD2 Is a Valuable Predictor Assessing Treatment Outcome in Acute Myeloid Leukemia,” Journal of Cancer 8, no. 3 (2017): 460–468. 28261348 10.7150/jca.16914 PMC5332898

[55] J. Quandt , S. Arnovitz , L. Haghi , et al., “Wnt‐β‐catenin Activation Epigenetically Reprograms T(reg) Cells in Inflammatory Bowel Disease and Dysplastic Progression,” Nature Immunology 22, no. 4 (2021): 471–484. 33664518 10.1038/s41590-021-00889-2 PMC8262575

[56] S. Keerthivasan , K. Aghajani , M. Dose , et al., “β‐Catenin Promotes Colitis and Colon Cancer Through Imprinting of Proinflammatory Properties in T Cells,” Science Translational Medicine 6, no. 225 (2014): 225ra28. 10.1126/scitranslmed.3007607 PMC4020714 24574339

[57] X. Li , Y. Xiang , F. Li , C. Yin , B. Li , and X. Ke , “WNT/β‐Catenin Signaling Pathway Regulating T Cell‐Inflammation in the Tumor Microenvironment,” Frontiers in Immunology 10 (2019): 2293. 31616443 10.3389/fimmu.2019.02293 PMC6775198

[58] M. A. Mazzola , R. Raheja , G. Murugaiyan , et al., “Identification of a Novel Mechanism of Action of Fingolimod (FTY720) on Human Effector T Cell Function Through TCF‐1 Upregulation,” J Neuroinflammation 12 (2015): 245. 26714756 10.1186/s12974-015-0460-z PMC4696082

[59] R. Firestein , A. J. Bass , S. Y. Kim , et al., “CDK8 is a Colorectal Cancer Oncogene That Regulates Beta‐Catenin Activity,” Nature 455, no. 7212 (2008): 547–551. 18794900 10.1038/nature07179 PMC2587138

[60] E. J. Morris , J. Y. Ji , F. Yang , et al., “E2F1 Represses Beta‐Catenin Transcription and Is Antagonized by both pRB and CDK8,” Nature 455, no. 7212 (2008): 552–556. 18794899 10.1038/nature07310 PMC3148807

[61] G. Tangherlini , D. V. Kalinin , D. Schepmann , et al., “Development of Novel Quinoxaline‐Based κ‐Opioid Receptor Agonists for the Treatment of Neuroinflammation,” Journal of Medicinal Chemistry 62, no. 2 (2019): 893–907. 30543421 10.1021/acs.jmedchem.8b01609

[62] K. L. Park , N. Y. Ko , J. H. Lee , et al., “4‐Chlorotetrazolo[1,5‐a]Quinoxaline Inhibits Activation of Syk Kinase to Suppress Mast Cells in Vitro and Mast Cell‐mediated Passive Cutaneous Anaphylaxis in Mice,” Toxicology and Applied Pharmacology 257, no. 2 (2011): 235–241. 21958720 10.1016/j.taap.2011.09.009

[63] T. Morokata , K. Ida , and T. Yamada , “Characterization of YM‐90709 as a Novel Antagonist Which Inhibits the Binding of Interleukin‐5 to Interleukin‐5 Receptor,” International Immunopharmacology 2, no. 12 (2002): 1693–1702. 12469943 10.1016/s1567-5769(02)00191-1

[64] P. Giusti , G. Frascaroli , C. Tammik , S. Gredmark‐Russ , C. Söderberg‐Nauclér , and S. Varani , “The Novel Anti‐Rheumatic Compound Rabeximod Impairs Differentiation and Function of Human Pro‐Inflammatory Dendritic Cells and Macrophages,” Immunobiology 216, no. 1‐2 (2011): 243–250. 20494473 10.1016/j.imbio.2010.04.004

[65] K. Lahl , C. Loddenkemper , C. Drouin , et al., “Selective Depletion of Foxp3+ Regulatory T Cells Induces a Scurfy‐Like Disease,” Journal of Experimental Medicine 204, no. 1 (2007): 57–63. 17200412 10.1084/jem.20061852 PMC2118432

[66] M. Lisurek , B. Rupp , J. Wichard , et al., “Design of Chemical Libraries With Potentially Bioactive Molecules Applying a Maximum Common Substructure Concept,” Mol Divers 14, no. 2 (2010): 401–408. 19685275 10.1007/s11030-009-9187-z PMC7089384

[67] S. I. Shulga and O. S. Shulga , “Synthesis and some Reactions of 6H‐Indolo[2,3‐b]Quinoxalines,” Russian Journal of Organic Chemistry 56, no. 12 (2020): 2104–2108.

[68] Y. Shimazu , Y. Shimazu , M. Hishizawa , et al., “Hypomethylation of the Treg‐Specific Demethylated Region in FOXP3 Is a Hallmark of the Regulatory T‐cell Subtype in Adult T‐Cell Leukemia,” Cancer immunology research 4, no. 2 (2016): 136–145. 26681759 10.1158/2326-6066.CIR-15-0148

[69] ggpubr: ‘ggplot2’ Based Publication Ready Plots [Internet]. 2023, https://rpkgs.datanovia.com/ggpubr/.

[70] K. J. Livak and T. D. Schmittgen , “Analysis of Relative Gene Expression Data Using Real‐Time Quantitative PCR and the 2(‐Delta Delta C(T)) Method,” Methods (San Diego, California) 25, no. 4 (2001): 402–408. 10.1006/meth.2001.1262 11846609

