# Supplementary material for: Generation of Regulatory T Cells From Human Memory CD4+T Cells by Upregulation of Naked Cuticle Homolog 2
Source: Eur J Immunol. 2025 Aug 5;55(8):e70018. doi: 10.1002/eji.70018 (PMC12322872; doi:10.1002/eji.70018)
Supplement: Supplementary file 1 — Supporting file 1: eji70018‐sup‐0001‐SuppMat.pdf [file EJI-55-e70018-s001.pdf]

Supplementary Material

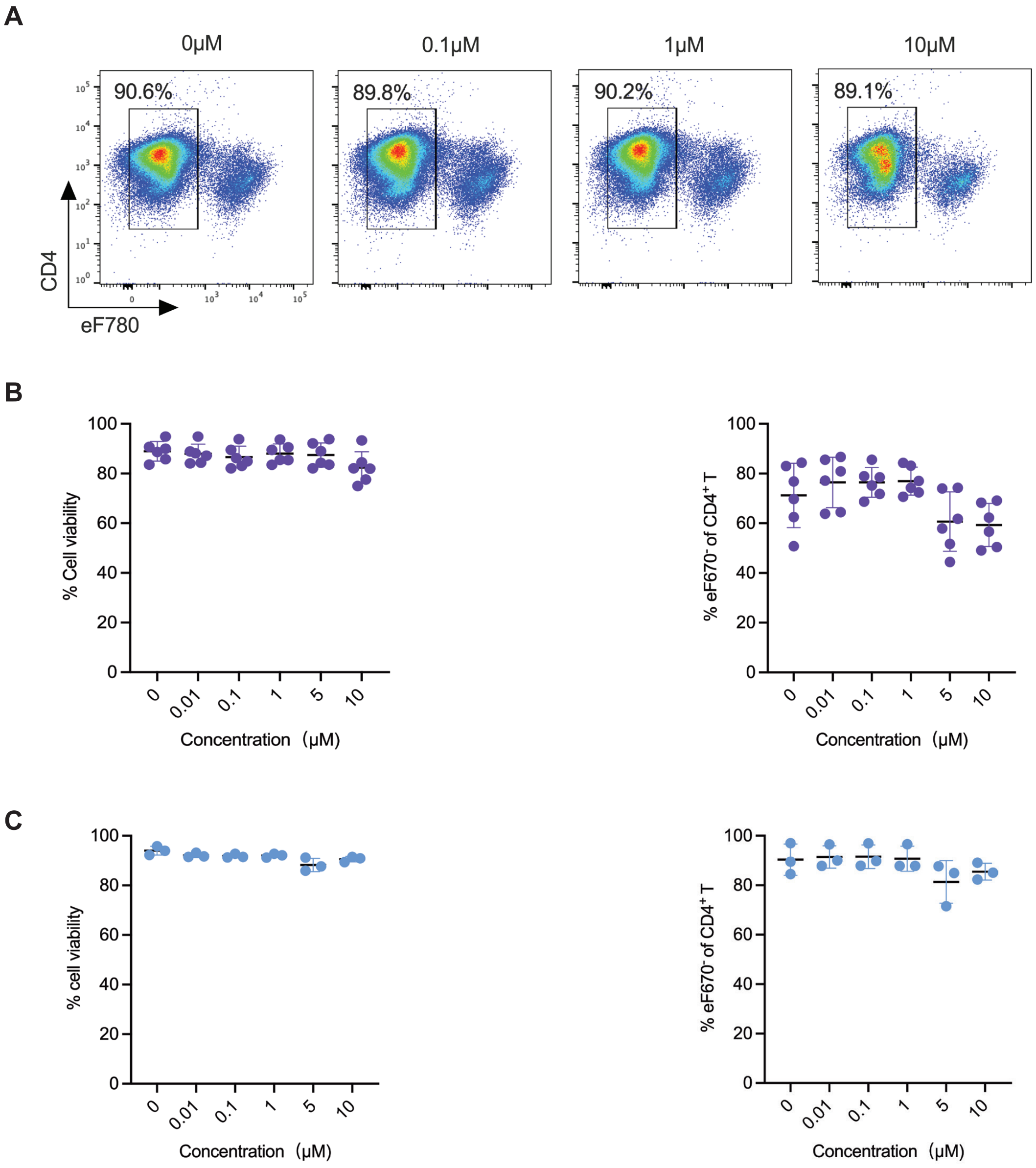

Figure S1: **Effects of IFA005 on the viability and proliferation of CD4^{+}T cells**

Tmem and T_N were stimulated with plate-bound anti-CD3/CD28 mAb in the presence of indicated concentrations of IFA005 for 5 days separately. The percentage of live cells and proliferated cells within memory and naïve CD4^{+}T were assessed by flow cytometry using fixable viability dye eFluor 780 (eF780) and cell proliferation dye eFluor 670 (eF670). (A) Representative flow dot plots show the viability of Tmem under various concentrations of IFA005. (B) The percentage of viability and proliferation of Tmem (n = 6). (C) Cell viability and proliferation of T_N (n = 3). All data are presented as mean ± SD, one-way ANOVA was performed for statistics, data points in B-C represent individual experiments.

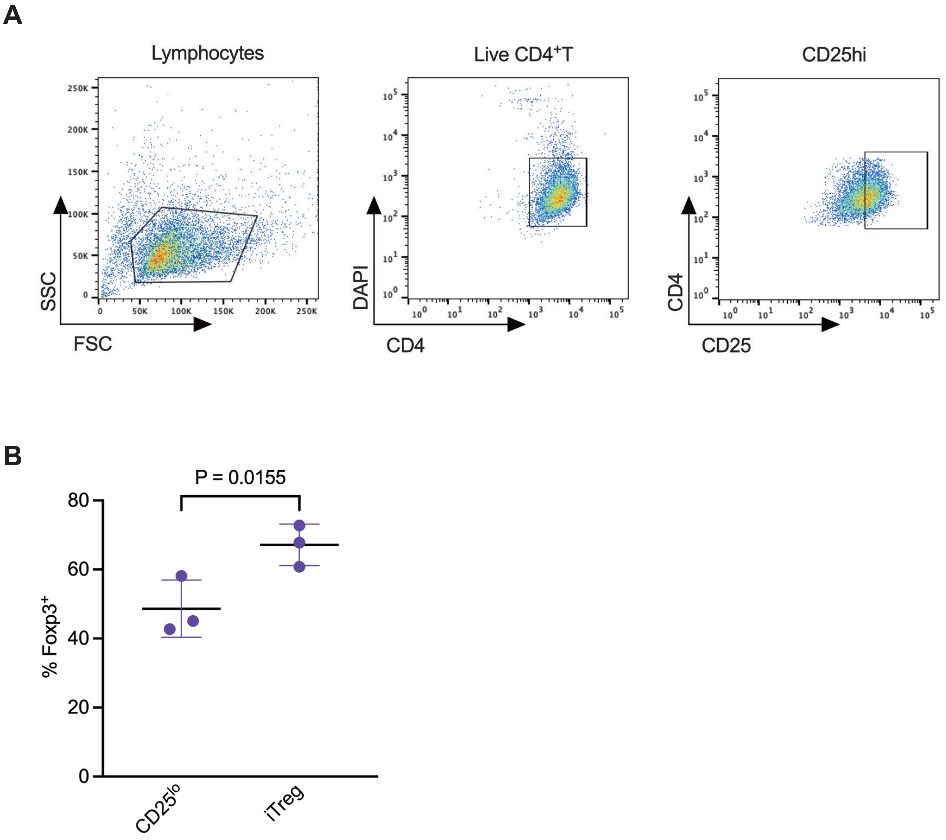

Figure S2: Sorting strategy and frequencies of Foxp3^{+} cells within IFA005-iTreg CD25^{high} versus CD25^{low}-expressing T cells.

Tmem were stimulated with plate-bound anti-CD3/CD28 mAb in the presence of IFA005 (1 μM) for 3 days. iTreg (the top 50% of CD25-expressing cells) and CD25^{low}-expressing (the bottom 30% of CD25-expressing cells) T cells were sorted, followed by Foxp3 measurement via flow cytometry. (A) The scheme for sorting strategy. (B) The percentage of Foxp3 expression, data were generated from 3 individual experiments and presented as mean ± SD, paired t-test was applied for statistics.

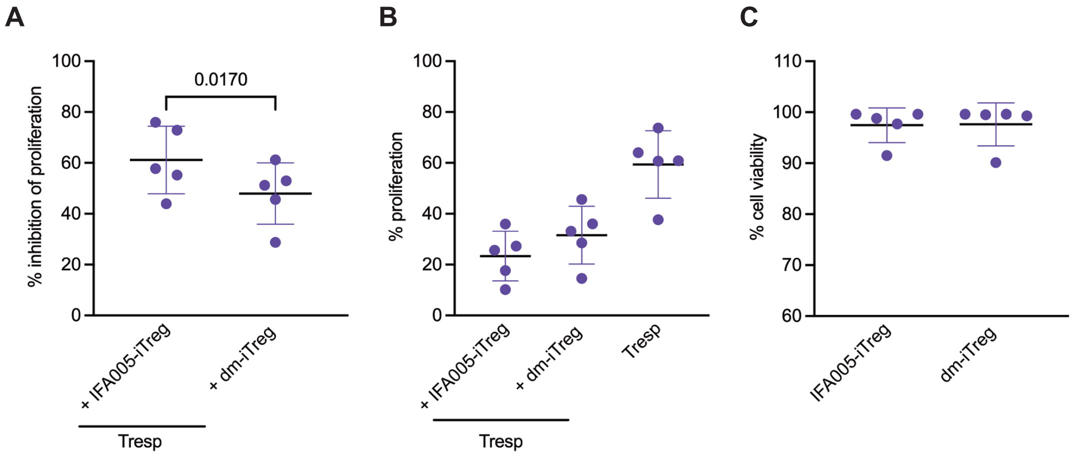

Figure S3: Sustained suppressive capacity of IFA005-iTregs.

Sorted IFA005-iTregs or dm-iTregs were cultured for 3 days in medium (X-VIVO 15 supplemented with penicillin/streptomycin, 10% FBS, and 100 IU/mL IL-2) without IFA005, then co-cultured with freshly isolated autologous CFSE-labeled $CD4^{+}CD25^{-}$ responder T cells (Tresp) in the presence of Treg Suppression Inspector for 72 hours. Shown are the percentage of inhibition (A) in the presence of the indicated iTregs at a 1:1 ratio, the corresponding percentage of Tresp proliferation (B), and the viability of iTregs (C) after the 3-day culture period. All conditions were performed in duplicates or triplicates. Data represent mean ± SD from 5 independent experiments. Statistical analysis was performed using a paired t-test.

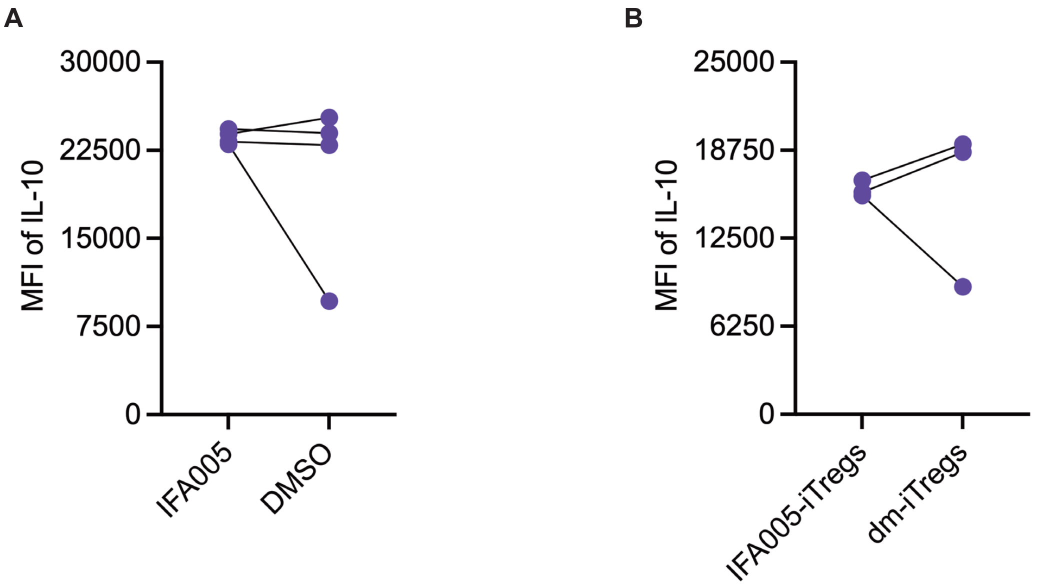

Figure S4: The secretion of IL-10 in supernatants of Tmem and by IFA005-iTreg

(A) IL-10 level in the supernatants of Tmem. Tmem was stimulated with plate-bound anti-CD3/CD28 mAb for 3 days in the presence or absence of IFA005 (1μM), supernatants were collected and measured (n = 4), Dimethyl sulfoxide (DMSO) was used as a mock. (B) The level of IL-10 secreted by iTregs. IFA005-iTregs and dm-iTregs were generated as mentioned in the method section and then stimulated with PMA/Iono for 5 hours, supernatants were collected and measured (n = 3). The MFI is a raw data readout of the Luminex instrument is shown. All data were presented as mean ± SD, paired t-test was applied for statistics, all data points represent individual experiments.

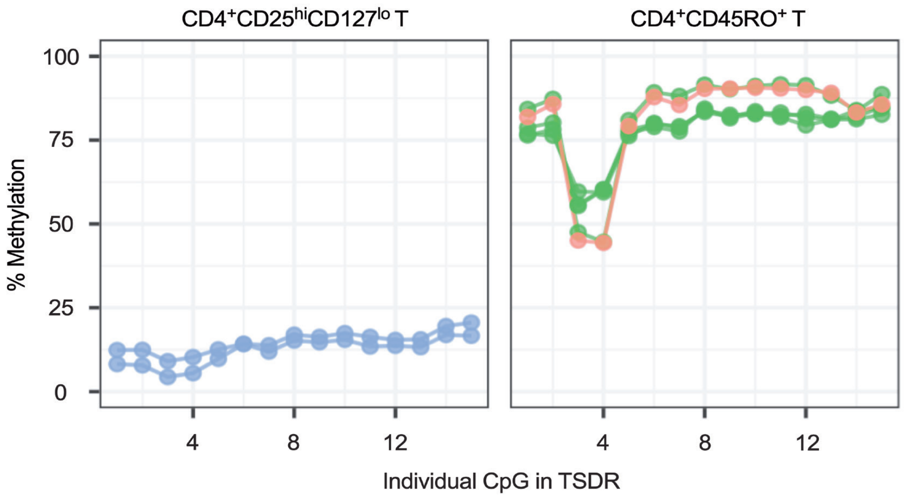

Figure. S5: CpG methylation status of the FOXP3-TSDR in IFA005-iTregs, tTregs and dm-iTregs.

The DNA methylation status of the FOXP3-TSDR was measured by bisulfite amplicon sequencing for all three analyzed populations. The plots show the methylation degree (on the Y axis, in %) for each individual CpG site within the FOXP3-TSDR (15 in total, displayed on the X axis). Green dots represent IFA005-iTregs, red dots represent dm-iTregs, blue dots represent tTregs.

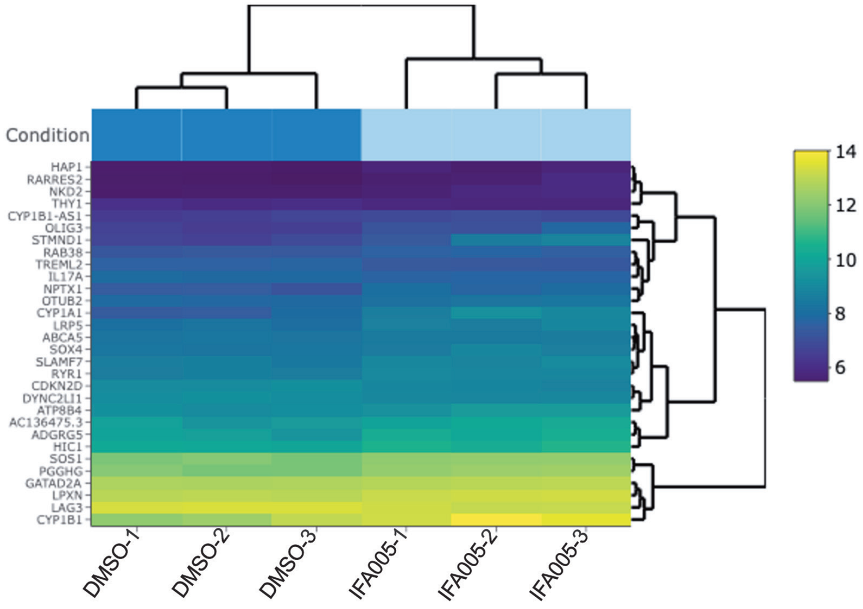

Figure. S6: The top 30 differentially expressed genes.

Hierachical-clustering heatmap displayed the expression profile of the top 30 differentially expressed genes sorted by their adjusted p-value by plotting their log2 transformed expression values in individual samples.

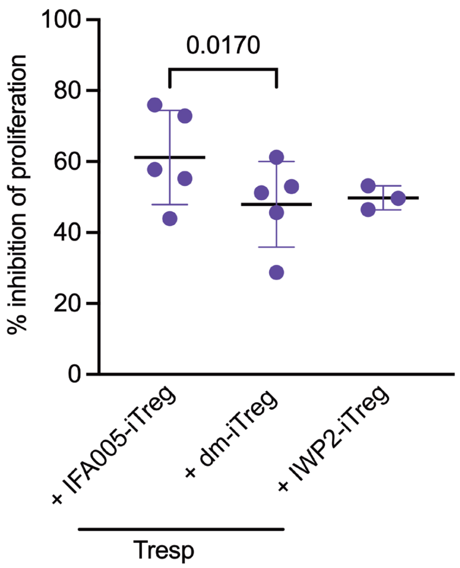

Figure S7: Comparative analysis of suppressive capacity of IFA005-iTregs and IWP2-treated cells.

IWP2-iTregs were generated using the same protocol as IFA005-iTregs and dm-iTregs. Specifically, Tmem were stimulated with plate-bound anti-CD3/CD28 antibodies in the presence of IWP2 (2 µM) for 3 days. The top 50% of CD25-expressing cells were then sorted and defined as IWP2-iTregs. All iTreg populations were cultured for an additional 3 days in the absence of the respective compounds before being co-cultured with freshly isolated, CFSE-labeled autologous Tresp at a 1:1 ratio in the presence of Treg Suppression Inspector for 72 hours. Shown is the percentage inhibition of Tresp proliferation by the indicated iTreg populations. dm-iTregs (DMSO-treated controls) were included as a reference. All conditions were performed in duplicates or triplicates. Data represent mean ± SD from 5 independent experiments, except for IWP2-iTregs, which are based on 3 independent experiments. Statistical analysis was performed using a paired *t*-test.

Table S1. Antibodies and reagents

	SOURCE	IDENTIFIER
**Antibodies**		
anti-human CD127 APC	Miltenyi Biotec	Cat#130-113-413
anti-human CD25 PE	Miltenyi Biotec	Cat# 130-113-286
Anti-human CD278 BB515	BD	Cat# 564549
anti-human CD39 BV510	BD	Cat# 567526
anti-human CD4 BV510	Biolegend	Cat# 317444
anti-human CD4 Percp	Miltenyi Biotec	Cat# 130-113-217
anti-human CD45RA PE-Vio 770	Miltenyi Biotec	Cat# 130-113-367
anti-human CD45RO VioGreen	Miltenyi Biotec	Cat# 130-113-561
anti-human CTLA-4 APC	Biolegend	Cat# 369612
anti-human Foxp3 PE	Thermo Scientific	Cat# 12-4777-42
anti-NKD2	Cell Signaling Technology	Cat# 2073S
anti-pGSK-3β AF647	Cell Signaling Technology	Cat# 14332S
anti-rabbit IgG AF647	Cell Signaling Technology	Cat# 4414S
anti-human TIGIT BV421	Biolegend	Cat# 372710
anti-β-Catenin AF647	Cell Signaling Technology	Cat# 4627S
anti-human CD127 APC	Miltenyi Biotec	Cat# 130-113-413
anti-human CD25 PE	Miltenyi Biotec	Cat# 130-113-286
anti-mouse CD4	In house	
StemMACS IWP-2	Miltenyi Biotec	Cat# 130-105-335

Ionomycin	Sigma-Aldrich	Cat# I3909-1ML
PMA	Sigma-Aldrich	Cat# P1585-1MG
X-VIVO15 medium	Lonza	Cat# 02-060F
DMSO (Dimethyl Sulfoxide)	SIGMA	Cat# D2650-100ML
2-propanol (IPA)	Carl Roth	Cat# CP41.1
4% Formaldehyde	Cell Signaling Technology	Cat# 47746S
Fetal Bovines Serum	Bio&Sell	Cat# FBS.S 0615 HI
Penicillin-Streptomycin Solution	Hyclone	Cat# SV30010
Cell Proliferation Dye eFluor 670	Thermo Fisher Scientific	Cat# 65-0840-85
CFSE	Thermo Fisher Scientific	Cat# 65-0850-84
Fixable Viability Dye eFluor™ 780	Thermo Fisher Scientific	Cat# 65-0865-14
Purified anti-human CD3 Antibody	Biolegend	Cat# 317302
Purified anti-human CD28 Antibody	Biolegend	Cat# 302902
SYBR Green Supermix	Bio-Rad	Cat# 1725271
Treg suppression Inspector	Miltenyi Biotec	Cat# 130-092-909
Human IL-2	Miltenyi Biotec	Cat# 130097743
Mouse IL-2	R&D systems	402-ML-100/CF
Mouse TGF-beta 1	R&D systems	Cat# 7666-MB
Ovalbumin	In house	

Memory CD4$^+$T cell isolation Kit,human	Miltenyi Biotec	Cat# 130-091-893
Naive CD4$^+$T cell isolation KIt II,human.	Miltenyi Biotec	Cat# 130-094-131
CD4$^+$T isolation Kit human	Miltenyi Biotec	Cat# 130-096-553
NucleoSpin RNA Set	Macherey Nagel	Cat# 740406.50
Foxp3/Transcription Factor Staining Buffer Set	Thermo Scientific	Cat# 00-5523-00
45-Plex Human ProcartaPlex™ Panel 1	Thermo Scientific	Cat# EPX450-12171-901
EZ DNA Methylation Kit	Zymo Research	Cat# D5001
ssDNA Assay-Kit	Thermo Fisher Scientific	Cat# Q10212
mRNA Purification Kit	Thermo Fisher Scientific	Cat# 61006
PrimeScript RT Reagent Kit	Takara Bio	Cat# RR037A
NKD2<br>Fwd-5’-CGGGATTGAGAACTACACGTC<br>Rev-5’-GGTGTACGGCATGTGTATCTG	TIB Molbiol	N/A
GAPDH<br>Fwd-5’- ACATCGCTCAGACACCATG<br>Rev-5’- TGTAGTTGAGGTCAATGAAGGG	TIB Molbiol	N/A
